# Retroperitoneal Extrusion of a Staghorn Calculus With a Nephrocutaneous Fistula Following a Percutaneous Nephrostomy: A Case Report and Literature Review

**DOI:** 10.7759/cureus.63136

**Published:** 2024-06-25

**Authors:** Mohamed S Mohsin, Nader F Gaballa, Shankar Chandrasekharan, Christopher J Dowson

**Affiliations:** 1 Urology, University Hospitals Birmingham NHS Foundation Trust, Birmingham, GBR

**Keywords:** spontaneous extrusion, nephrostomy, nephrocutaneous fistula, nephrolithiasis, staghorn calculus, urolithiasis

## Abstract

Renal calculi forming a nidus for chronic infection is an established cause of nephrocutaneous fistulation. Although uncommon, extrusion of renal calculi from the kidney can occur on rare occasions.

We describe a case of a spontaneously extruded staghorn calculus measuring 3.5 x 2.5 cm from the kidney into the retroperitoneal space resulting from a neglected nephrostomy tube resulting in a nephrocutaneous fistula. We describe the surgical management of the extruded calculus.

## Introduction

Nephrolithiasis is a common urological condition affecting 12% of adults during their lifetime [1]. Management depends on the nature of the presentation. In the context of obstructing stones or infected calculi, emergency intervention in the form of renal decompression utilising a nephrostomy tube or ureteric stent is indicated.

In the non-emergency setting, factors such as the location of the calculus, size, stone factors, patient factors, patient preference, and fitness for surgery are important considerations to achieve a satisfactory stone-free rate.

Staghorn calculi are a form of renal calculi that branch into the renal calyces and account for up to 20% of all urinary tract calculi historically. However, in the developed world, the incidence of this form of urolithiasis stands at 4% [2]. Spontaneous extrusion of urinary calculi is very rare.

We present a case report of a spontaneous extrusion of calculus into the retroperitoneum with a resulting nephrocutaneous fistula secondary to both chronic infection and a pre-existing nephrostomy tract. The calculus was managed with a combination of endourological, open extraction of the calculus, and minimally invasive radiologically guided interventions.

## Case presentation

A 48-year-old lady with a history of intravenous drug and substance abuse was referred to the department of urology in 2014 with an obstructing 6-mm calculus in the right vesicoureteral junction. Although the patient was afebrile with a blood pressure of 140/95 mmHg and a heart rate of 106 beats per minute, she had a raised white cell count of 16.1 x 109/L and a C-reactive protein level of 139 mg/L (0-5 mg/L) with a creatinine of 108 umol/L (49-90 umol/L) and an estimated glomerular filtration rate of 48 ml/min/1.73 m^2^ (>90 ml/min/1.73 m^2^), in addition to being peripherally shut down with difficult venous access, pain, and vomiting. With the concern being urosepsis, a nephrostomy tube was inserted to avoid delays and the progression to septic shock while awaiting retrograde ureteric stent insertion in the operating theatre. Following the insertion of the percutaneous nephrostomy tube, an elective ureteroscopy (URS) with laser lithotripsy was scheduled. The procedure was initially deferred due to a new diagnosis of hypertension requiring medical optimisation. The patient was uncontactable for five months following the deferred URS - the first of multiple occasions our patient was unable to attend an appointment with the department.

**Figure 1:**
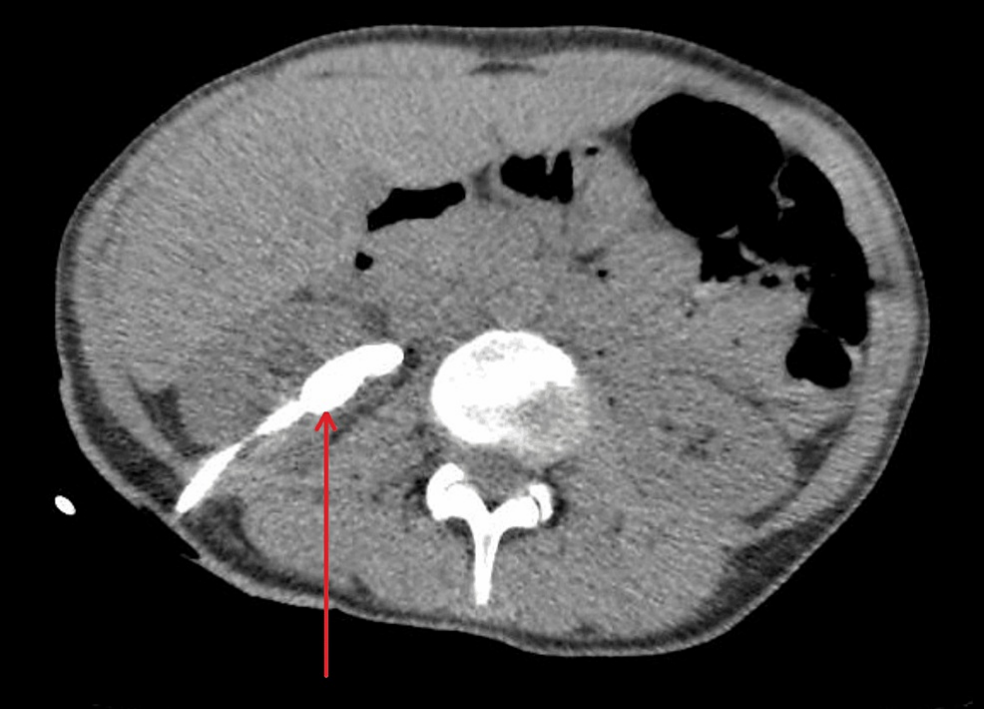
Non-contrast CT scan of the kidneys demonstrating an encrusted coil of the nephrostomy tube within the right kidney (red arrow).

Nine months following the insertion of the nephrostomy, a non-contrast CT of the urinary tract (NCCT-KUB) demonstrated that the distal ureteric calculus had passed. However, the nephrostomy tube was encrusted within a 3.3 x 1.7 cm stone (Figure 1). The kidney was unobstructed. The patient was scheduled for percutaneous nephrolithotomy (PCNL) but failed to attend her follow-up appointments.

**Figure 2:**
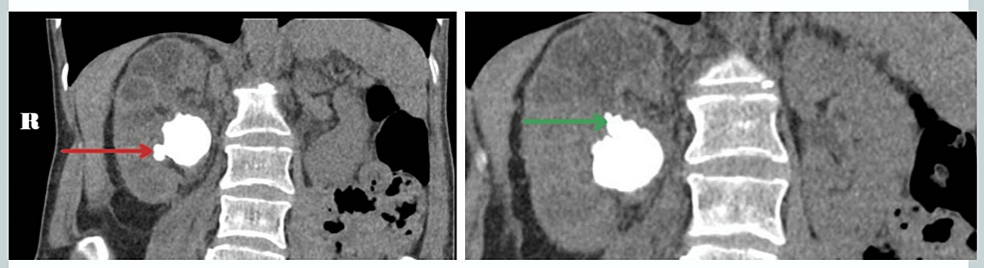
Pre-contrast image demonstrating a staghorn calculus within the right kidney. The calculus is seen to branch into the lower calyx (red arrow) and the upper calyx (green arrow).

In 2017, 38 months after the insertion of the nephrostomy, the patient was admitted with right flank pain and a blocked nephrostomy. A contrast-enhanced CT of the urinary tract (CECT-KUB, Figure 2) demonstrated a staghorn calculus inseparable from the nephrostomy tube measuring 3.5 x 2.4 cm and changes keeping with xanthogranulomatous pyelonephritis (XPN). A dimercaptosuccinic acid (DMSA) scan yielded a shrunken right kidney contributing 15% of the overall split function. Therefore, an elective nephrectomy was planned following the optimisation of her nutritional status under the care of a dietician.

In 2018, the patient accidentally pulled out the external portion of the nephrostomy tube. An NCCT-KUB demonstrated that the stone had marginally enlarged to 3.8 x 2.3 cm with XPN, in addition to two new mid-ureteric calculi, each measuring 5 mm without associated hydroureter. A small subcutaneous tract with surrounding enhancement was also observed in the posterolateral right side of the abdomen.

The patient did not attend planned follow-up appointments until her admission to the hospital in the summer of 2023 with right loin pain and intermittent purulent discharge from the site of her nephrostomy tube. On examination, the patient was tachycardic and afebrile with purulent discharge from the fistula on her right flank.

**Figure 3:**
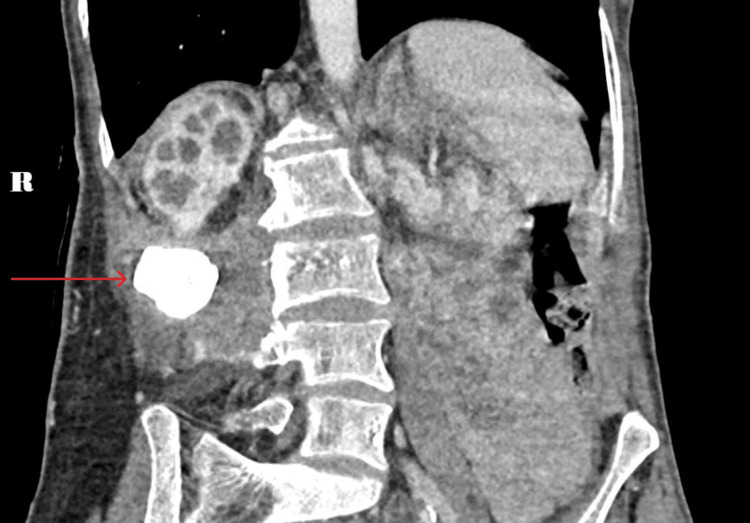
Migrated calculus within the right retroperitoneum abutting the iliopsoas (red arrow).

**Figure 4:**
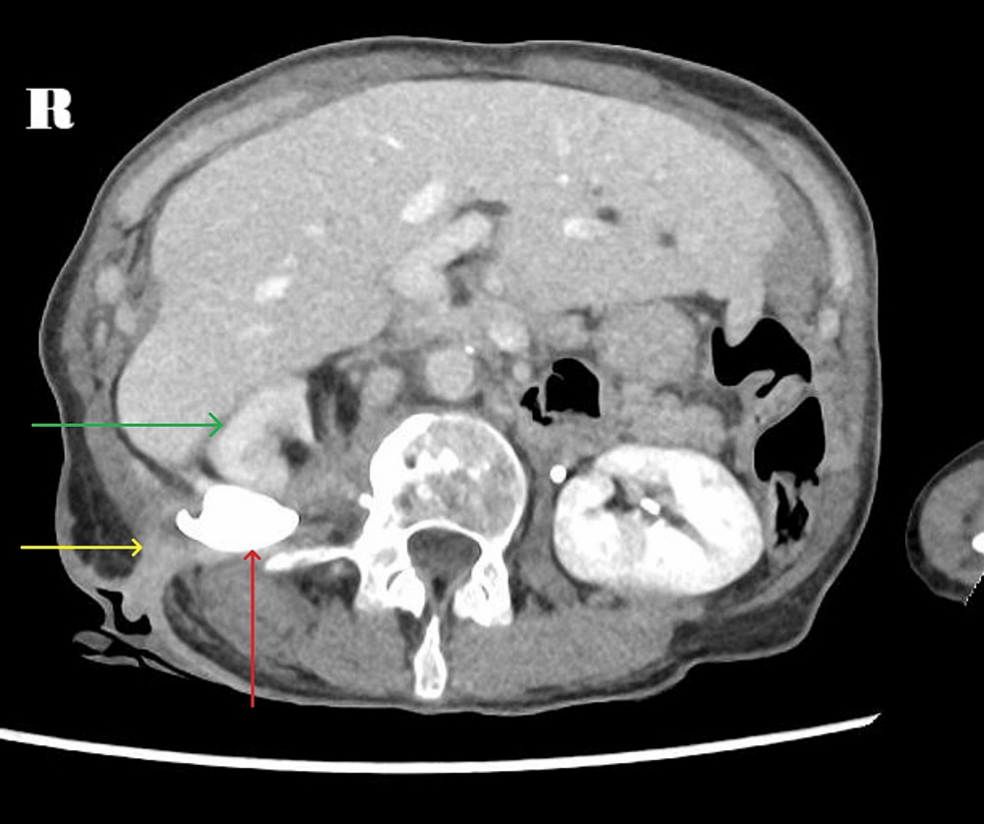
Nephrocutaneous fistula (yellow arrow) with the calculus lying within the retroperitoneum (red arrow) with the lack of contrast in the right collecting system and poor enhancement of the renal parenchyma (green arrow).

An NCCT-KUB demonstrated that the previous right renal pelvic stone had migrated posteriorly, out of Gerota’s fascia to lie along the quadratus lumborum with a surrounding organised collection containing air loculi abutting the iliopsoas muscle (Figure 3). A CECT-KUB confirmed a cutaneous fistula with a urine leak into the collection adjacent to the migrated staghorn calculus with no discernible excretion of contrast into the right ureter (Figure 4).

**Figure 5:**
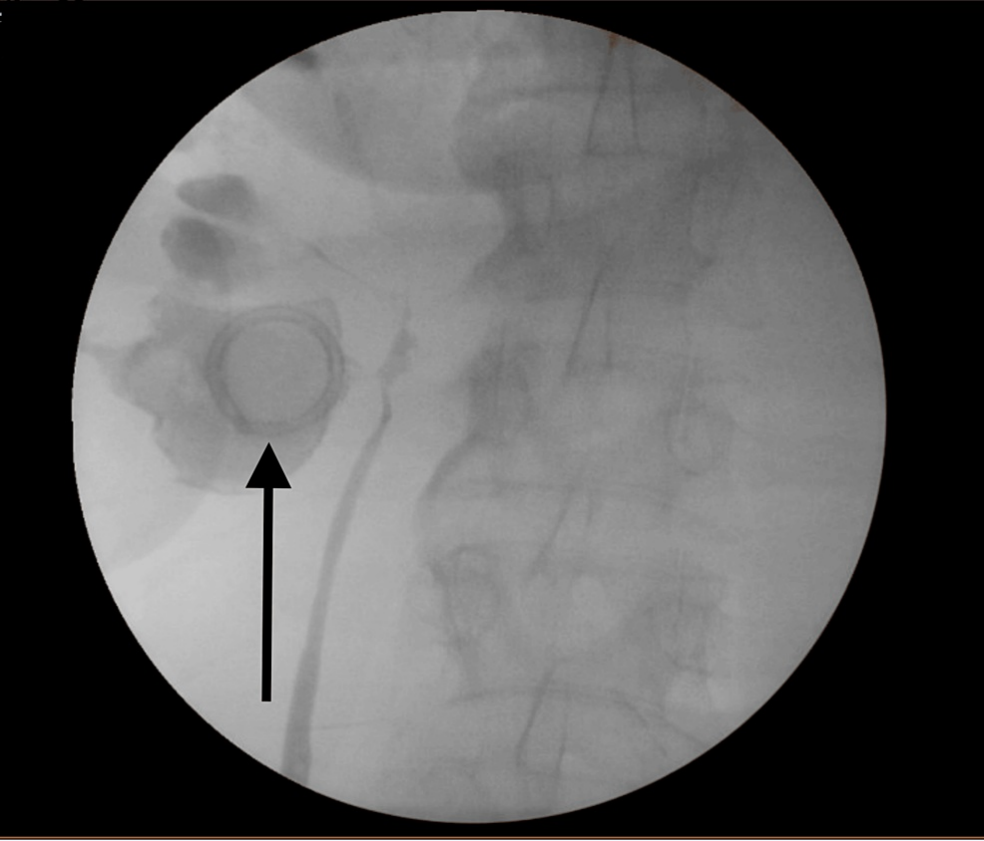
Retrograde uretero-pyelogram demonstrating a narrow pelvi-ureteric junction with the encrusted coil of the nephrostomy tube within the right kidney (black arrow).

The patient was frail and malnourished with an Eastern Cooperative Oncology Group (ECOG) performance status of 2 and considered high risk for a nephrectomy. Instead, the patient underwent a right retrograde uretero-pyelogram and ureteric stent insertion. Apart from a narrow pelvi-ureteric junction negotiated with a guidewire, the stone was otherwise evident with minimal extravasation of contrast appreciated (Figure 5). A 6 Fr/26 cm ureteric stent was inserted.

**Figure 6:**
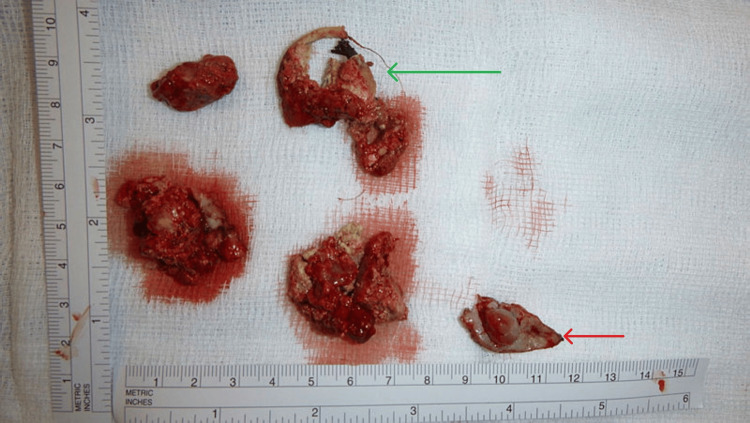
The coil of the encrusted nephrostomy tube (green arrow) and three fragments of the calculus, which disintegrated following extraction. Excised skin overlying the nephrostomy tract (red arrow).

Next, the patient was re-positioned in the kidney rest left lateral decubitus position with a table break similar to an open or laparoscopic approach to the kidney. An elliptical incision was made over the nephrostomy tract and the dissection was continued through the indurated layers until the stone was visualised, which was fairly superficial given the patient's thin frame. The intact stone and the tip of the nephrostomy tube were removed (Figure 6), followed by a thorough washout with 0.9% sodium chloride, the insertion of a passive Robinson drain, and the alginate packing of the wound.

Five days post operation, a CECT-KUB showed an organised collection with the surgical drain within the collection. The proximal tip of the ureteric stent was posterior and inferior to the right renal pelvis and proximal ureter, which was attributed to a friable renal pelvis and proximal ureter. A percutaneous nephrostomy tube was inserted, and the ureteric stent was removed.

The nephrostomy tube was displaced following insertion and the absence of pelvicalyceal dilatation made its reinsertion technically impossible despite efforts by the interventional radiologists. A CECT-KUB 14 days post operation demonstrated an atrophic, moderately hydronephrotic right kidney with contrast draining into the bladder.

After a period of observation, the drain output was minimal and subsequently removed. The patient made a satisfactory recovery in the hospital before discharge with no morbidity or mortality reported in a period of six months post operation.

## Discussion

Spontaneous extrusion of renal calculus is a rare occurrence described previously in only five case reports. An early case report by Breatnach et al. (1986) [3] reported the CT finding of an extruded staghorn calculus into the flank soft tissue. Lewi and Scott (1986) [4] observed three common elements among patients: a perinephric abscess, a staghorn calculus in a non-functioning kidney, and a fragment of stone separate from the main bulk, with seven cases reported at the time. Vaidyanathan et al. (2001) [5] reported spontaneous extrusion of a staghorn calculus in a patient with spinal cord injury due to delayed diagnosis with extrusion ex vivo not requiring surgical intervention. Purkait et al. (2016) [6] reported a case of spontaneous extrusion of staghorn renal calculus with nephrocutaneous fistula in a child managed with a subcapsular nephrectomy, extraction of the impacted staghorn calculus embedded in the psoas muscle, and excision of the fistula. Chandrasekar et al. (2024) [7] reported a case of a surfacing staghorn, which was manually extracted at the patient’s bedside.

To the best of our knowledge, our patient is the first case of an established nephrostomy tract predisposing to the extrusion of the calculus. The current standard of nephrostomy care involves replacement every three months. We conclude that the nephrostomy tube acted as a nidus for encrustation with deposits of urinary salts leading to gradual enlargement of the calculus and resulting in a staghorn calculus.

Bacterial cultures obtained at the time of the operation confirmed the presence of *Proteus mirabilis*, a urea-splitting organism, which can lead to the formation of struvite stones [8,9]. In a study of complete staghorn calculi conducted by Viprakasit et al. (2011) [10], it was found that 44% of the patients had developed infection-related staghorn calculi with the remainder being metabolic in origin.

Recurring cycles of infection, stone growth, and the existing nephrocutaneous fistula provided a conduit for purulent discharge to drain out of the kidney with ensuing parenchymal thinning eventually leading to the extrusion of the staghorn calculus in toto into the retroperitoneum.

## Conclusions

The extrusion of a staghorn calculus is a rare occurrence. The patient in this case had a history of a staghorn calculus forming secondary to an encrusted nephrostomy tube with the ultimate development of a nephrocutaneous fistula. The authors propose that the recurrent infection coupled with the fistula, atrophy, and non-functional nature of the kidney lead to a path of least resistance forming with subsequent expulsion of the stone into the retroperitoneal space.

Poor patient compliance and lifestyle factors hampered interventions planned earlier on several occasions. A nephrectomy may be considered definitive management in such cases but given the co-morbidities in this specific patient, major surgery was considered very high risk. Therefore, the management was tailored to minimise the impact of surgery and, in this case, excision of the tract and extraction of the calculus with percutaneous drain insertion and urinary diversion with a ureteric stent in the first instance and later, a nephrostomy resulted in a satisfactory outcome.
